# Development, acceptability and feasibility of a personalised, behavioural intervention to prevent bacterial skin and soft tissue infections among people who inject drugs: a mixed-methods Person-Based Approach study

**DOI:** 10.1186/s12954-023-00823-9

**Published:** 2023-08-22

**Authors:** Joanna Kesten, Deborah Hussey, Catherine Lord, Leonie Roberts, James Bayliss, Helen Erswell, Andrew Preston, Maggie Telfer, Jenny Scott, Magdalena Harris, Dominic Mellon, Matthew Hickman, Georgie MacArthur, Harriet Fisher

**Affiliations:** 1 Population Health Sciences, Bristol Medical School, University of Bristol, Bristol, UK; 2 The National Institute for Health and Care Research (NIHR) Health Protection Research Unit (HPRU) in Behavioural Science and Evaluation, University of Bristol, Bristol, UK; 3 The National Institute for Health and Care Research Applied Research Collaboration West (NIHR ARC West), University Hospitals Bristol and Weston NHS Foundation Trust, Bristol, UK; 4 Bristol Drugs Project, Bristol, UK; 5 Bristol City Council, Bristol, UK; 6 Bristol, North Somerset, South Gloucestershire Integrated Care System, Bristol, UK; 7 UK Health Security Agency (UKHSA), South West Region, Bristol, UK; 8 Exchange Supplies, Dorchester, UK; 9 Centre for Academic Primary Care, Bristol Medical School, University of Bristol, Bristol, UK; 10 London School of Hygiene and Tropical Medicine, London, UK

**Keywords:** People who inject drugs, Skin and soft tissue infections, Co-production, Person-Based Approach, Intervention development, Acceptability, Feasibility, Harm reduction, Behaviour change

## Abstract

**Background:**

Skin and soft tissue infections (SSTI) among people who inject drugs (PWID) are a public health concern. This study aimed to co-produce and assess the acceptability and feasibility of a behavioural intervention to prevent SSTI.

**Methods:**

The Person-Based Approach (PBA) was followed which involves: (i) collating and analysing evidence; (ii) developing guiding principles; (iii) a behavioural analysis; (iv) logic model development; and (v) designing and refining intervention materials. Co-production activities with target group representatives and key collaborators obtained feedback on the intervention which was used to refine its design and content. The intervention, harm reduction advice cards to support conversation between service provider and PWID and resources to support safer injecting practice, was piloted with 13 PWID by four service providers in Bristol and evaluated using a mixed-methods approach. Semi-structured interviews were conducted with 11 PWID and four service providers. Questionnaires completed by all PWID recorded demographic characteristics, SSTI, drug use and treatment history. Interviews were analysed thematically and questionnaires were analysed descriptively.

**Results:**

Published literature highlighted structural barriers to safer injecting practices, such as access to hygienic injecting environments and injecting practices associated with SSTI included: limited handwashing/injection-site swabbing and use of too much acidifier to dissolve drugs. Co-production activities and the literature indicated vein care and minimisation of pain as PWID priorities. The importance of service provider–client relationships and non-stigmatising delivery was highlighted through the co-production work. Providing practical resources was identified as important to address environmental constraints to safer injecting practices. Most participants receiving the intervention were White British, male, had a history of SSTI and on average were 43.6 years old and had injected for 22.7 years. The intervention was well-received by PWID and service providers. Intervention content and materials given out to support harm reduction were viewed positively. The intervention appeared to support reflections on and intentions to change injecting behaviours, though barriers to safer injecting practice remained prominent.

**Conclusions:**

The PBA ensured the intervention aligned to the priorities of PWID. It was viewed as acceptable and mostly feasible to PWID and service providers and has transferability promise. Further implementation alongside broader harm reduction interventions is needed.

## Background

Bacterial skin and soft tissue infections (SSTI) among people who inject drugs (PWID) such as abscesses and cellulitis are an increasing international public health concern [1–4]. SSTIs range from minor, self-limiting infections to life-threatening diseases requiring immediate medical care (e.g. endocarditis and deep vein thrombosis) [5]. While abscesses and cellulitis can be less serious, without treatment they can result in severe morbidity and mortality [6]. Treatment varies depending on the type of SSTI but typically involves cleaning and dressing of infection sites and antibiotics [4]. Although early medical treatment may prevent more serious consequences of infection, self-care is common [4].

SSTIs are common among PWID globally [1, 3, 4]. In England, Wales and Northern Ireland approximately 30% (95% CI 27–33%) of PWID report experiencing an abscess, sore or open wound at an injection site in the previous year [7]. Hospital admissions for PWID due to SSTI are also concerningly high [4] and have increased annually since 2012 in England [8]. The most common cause of SSTI is poor hygiene during injection site preparation [4]. Injection of drugs not manufactured for injection such as benzodiazepines and other pharmaceuticals, reuse of injecting equipment and unhygienic injecting environments are also causal mechanisms for SSTI [4]. SSTI among PWID are associated with older age, years injecting, more than one attempt to inject into the vein, injecting into the hands, feet, groin or neck and needle/syringe reuse or sharing [9].

Qualitative studies demonstrate that extreme pain caused by injecting-related injury [10] and SSTIs [11] can be normalised and seen as inevitable by PWID and delayed healthcare seeking is common [10]. One qualitative study found that the development of an SSTI can evoke panic and fear among PWID, as well as being stigmatising—both internally and socially—due to their visible physical symptoms which can identify an individual as someone who injects drugs [11]. PWID can feel a sense of personal responsibility for developing an SSTI, despite the social and physical environmental risks that are largely outside their control [11]. PWID awareness of SSTI typically originates from previous experience, rather than peer-to-peer information sharing, possibly due to the stigma of infections [11].

Opioid Agonist Treatment (OAT), which decreases injecting frequency and blood-borne virus transmission, is associated with some protection against SSTI hospitalisation or re-hospitalisation [2], and high OAT and needle and syringe programme (NSP) uptake in the past year are associated with a reduced risk of SSTI compared to low use of these harm reduction measures [12]. However, given the high levels of SSTI, additional harm reduction measures are also required. Other suggested strategies to prevent SSTI include provision of advice and information, coupled with interventions that reduce environmental or structural risk factors (e.g. the injecting environment) [11]. There have been suggestions for focussing interventions on pertinent and relevant issues to PWID such as venous access and care [9, 13] and pain associated with injecting and SSTI. However, there is currently limited evidence on effective interventions and most behavioural interventions have been conducted outside the UK [14–18].

In this paper, we aimed to report the process we followed to develop and assess the acceptability and feasibility of REACT (REducing bActerial infecTions), an individualised, behavioural, one-to-one pilot intervention to prevent bacterial SSTI among PWID consisting of a set of themed cards to facilitate positive, non-judgemental conversation between service providers and PWID.

## Methods

### Research setting

The study was undertaken in Bristol, the largest city in the South West of England where in 2020/2021 there were an estimated 4940 people who use opioids and/or crack cocaine, the second highest rate in English cities, including a high proportion of people with complex needs and an ageing population [19]. It is estimated that approximately half of these people inject drugs [20].

### Intervention planning

The intervention was conceived during the Local Government Association-funded, and Design Council-led ‘Design in the Public Sector’ (DiPS) programme (2018–2019). The DiPS programme aimed to improve capacity in the public sector to deliver efficient and effective services, while equipping local government with the knowledge and expertise to use and apply design principles in their day-to-day work. DiPS followed the Design Council’s Double Diamond Model [21] comprised of four phases. First, ‘Discover’ in which the problem of SSTI among PWID was looked at from a fresh perspective. Second, this information was used to further focus or ‘Define’ the problem. The third phase ‘Develop’ produced solutions to test and refine. Last, the ‘Deliver’ phase finalised these solutions into a project (e.g. product or service design/re-design). The programme was delivered rapidly over five one-day workshops and two coaching sessions with design experts. At the end of the programme, four priority areas had been developed: (1) improved infection prevention and control (e.g. consideration of injecting environments and the use of alcohol wipes prior to injection); (2) optimisation of harm reduction practice in the community; (3) increased access to healthcare among PWID (e.g. address negative perceptions of care and treatment in hospital, the hospital environment itself, attitudes and perceptions towards PWID among health professionals, and certain aspects of healthcare delivery, such as fast and appropriate OAT and harm reduction advice); and (4) improved adherence to treatment for SSTI (through peer and outreach support) [11, 22].

This paper addresses one workstream focused around priority area two: development of a brief individualised 1:1 motivational intervention to be delivered by a range of service providers who have regular contact with PWID and can therefore deliver the intervention opportunistically in practice (e.g. shared care workers who deliver OAT in partnership with general practitioners within primary care, pharmacists delivering OAT and NSP, hostel staff).

**Fig. 1:**
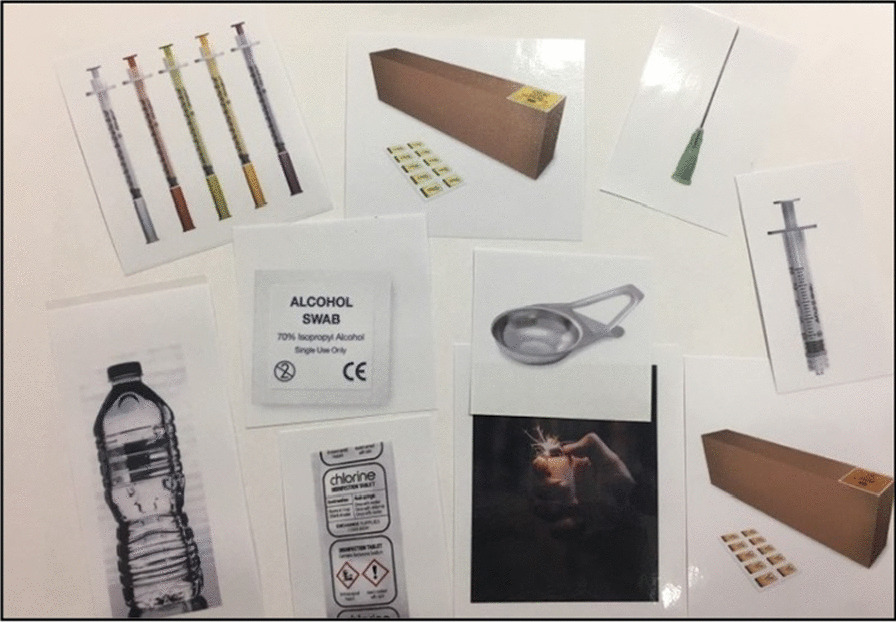
Prototype intervention materials

The DiPS programme co-developed a prototype for the intervention comprising a tailored conversation focussing on reducing risky injecting practices, self-care, sign-posting, and information relating to hospital admission. The prototype intervention consisted of a laminated set of images of injecting equipment and paraphernalia (Fig. 1) used to structure the 1:1 conversation about injecting practice and to identify areas where risk could be reduced. Details of locally available sources of healthcare were also included, alongside images of different stages of infection to support decisions around self-care and healthcare seeking.

Preliminary feedback from PWID and service providers suggested that the prototype intervention could be useful, acceptable, and feasible. However, feedback suggested a requirement to further optimise the prototype to be deliverable within settings used by PWID by service providers without specialist harm reduction knowledge and training to expand the reach of harm reduction advice. Funding was sought to enable further development of the intervention and piloting.

We then used the ‘Person-Based Approach’ (PBA) to intervention planning and development (https://www.personbasedapproach.org/) to refine and further develop the prototype intervention (described above). The PBA was followed to concentrate on the users experience, in turn increasing acceptability and effectiveness of resources. This approach involved combining stakeholder and Patient and Public Involvement (PPI) co-production with qualitative and mixed-methods research. Through in-depth understanding of the target group representatives’ perspectives, interventions can be designed or modified to ensure they are relevant, persuasive, accessible and engaging, and more successful to implement [23].

**Fig. 2:**
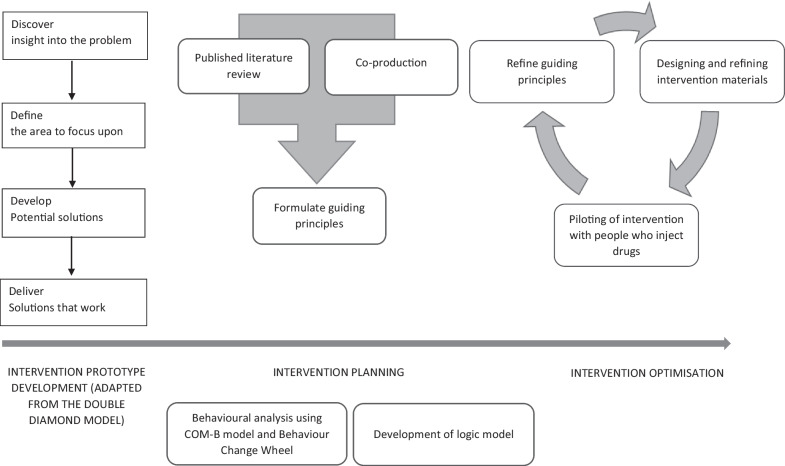
Intervention development overview. Adapted from the Person-Based Approach

Intervention development followed interrelated and iterative stages: (i) collating and analysing evidence; (ii) developing guiding principles; (iii) undertaking a behavioural analysis; (iv) developing a logic model; and (v) refining intervention materials. Figure 2 provides an overview of the process followed.

#### Collating and analysing evidence

##### Review of the relevant literature

A review of relevant literature was undertaken as recommended by the PBA. Two members of the research team (HF and JK) with input from academic experts collated qualitative, quantitative and mixed-methods, secondary research evidence that highlighted relevant behaviours and structural barriers to preventing bacterial SSTI. Additional hand searching of citations and reference lists supplemented the original evidence.

##### Co-production with target group representatives and key collaborators

We undertook co-production activities with 15 target group representatives in-person between February and March 2020, 6 service providers identified as potential intervention deliverers by telephone between May and June 2020 and 11 key collaborators (DH, CL, LR, JB, HE, AP, MT, JS, MH, DM, MH) during online meetings held between August 2020 and March 2021 [23]. This was considered appropriate (instead of undertaking an in-depth qualitative enquiry) because of the existing qualitative evidence base detailing the barriers and facilitators to safer injecting practice.

Key collaborators (DH and CL) with specialist expertise in delivering harm reduction advice in a drug and alcohol service undertook co-production activities with PWID. PWID with diverse experiences were sought from a range of settings including a city centre and satellite locations. PWID with a mix of genders, ages and accommodation statuses contributed. Most were White British, had been injecting for several years and reported experiences of SSTI. The co-production activities involved delivering and obtaining feedback on the process of delivery of the prototype intervention and detailing reflections of appropriateness, relevance and potential for intervention effectiveness using consistent templates. Researchers (JK and HF) produced a summary of the findings from the feedback which was used to refine the design of the intervention.

Co-production activities with service providers were undertaken by a researcher (HF) to gain understanding about how the intervention could be delivered in practice, using a topic guide focused on: (i) current knowledge about injecting behaviours, prevention of SSTI and services for PWID; (ii) intervention content and design; and (iii) hypothetical intervention delivery.

Finally, as the evidence was gathered, potential refinements of the intervention prototype were discussed with key collaborators, who included an assertive engagement worker within a drug and alcohol service (DH), the founder of a social enterprise that develops harm reduction equipment (AP), the chief executive of a drug and alcohol services charity (MT), multi-disciplinary academics (MH, JS), a pharmacist prescriber (JS) and public health and healthcare systems professionals (GM, JB, HE, DM).

##### Analysing the evidence from co-production activities

The purpose of this stage was not to undertake an in-depth qualitative enquiry but to inform intervention development. Findings and feedback from target group representatives and key collaborators were collated in an Intervention Planning Table [24, 25]. The possible implications and intervention features required to address the findings/feedback were documented. Changes were based on the MoSCoW prioritisation criteria (must have, should have, could have, would like) informed by the guiding principles [24, 25].

#### Developing guiding principles

Guiding principles are a key feature of the PBA and are intended to maximise the acceptability of the intervention and future engagement [24]. They comprise a design objective and proposed intervention features which address the user/context-specific behavioural needs. Provisional guiding principles were iteratively developed by the research team based on the knowledge gained from the review and consultation activities and refined as further understanding was gained throughout the study.

#### Undertaking a behavioural analysis

The behavioural analysis was informed by the literature review, co-production/consultation activities and intervention refinement process. It aimed to identify behaviours to be targeted by the intervention and their potential barriers and facilitators. These were mapped onto constructs from the Capacity, Opportunity, Motivation-Behaviour (COM-B) model of behaviour change and the Behaviour Change Wheel [26] to clearly describe the intervention processes and components, and behaviour change techniques [27]. The COM-B model offers a framework with which to design interventions with these key features of behaviour change in mind. The Behaviour Change Wheel was selected as it was designed to help researchers link behaviours to inform intervention design.

#### Logic model

A logic model was developed to provide a visual representation of the proposed mechanisms of change. This brings together the findings from the activities described above and how these are anticipated to reduce harm from SSTI among PWID.

### Intervention optimisation

#### Refining of intervention materials

We worked with a professional graphic designer specialising in harm reduction (Michael Linnell of Linnell Publications) to refine the intervention materials.

Feedback on the refined intervention materials was then obtained through additional co-production activities with key collaborators through online meetings and a new group of PWID by phone due to COVID-19 restrictions (n = 3). Feedback was elicited on their perceptions of the positive and negative aspects of the intervention materials, how it was presented, the design and suggestions for new content or messages. The responses were collated in a PBA Table of Changes document [24].

Modifications to the intervention materials were made in line with the guiding principles. This considered whether changes were likely to impact on behaviour change or a precursor to behaviour change (e.g. acceptability, feasibility, persuasiveness, motivation, engagement). Prioritisation for changes were based on the MoSCoW criteria [28].

Researchers (HF and JK) also developed a short intervention introduction manual containing information about the study, instructions for how to use the intervention materials, and a ‘questioning and resources’ guide to accompany it. The manual encouraged service providers to tailor the intervention to the client’s needs, the time available and the purpose of the conversation, emphasising that not everyone will necessarily benefit from every card. A short online course was developed for service providers to provide the necessary information and knowledge underpinning delivery of the intervention. This included an assessment of service provider knowledge. Key collaborators were invited to review and comment on the documents to ensure the content was accurate, evidence-based and consistent with best harm reduction practice.

#### Intervention pilot

Service providers were recruited through existing relationships within the project team and were provided with an information sheet describing the pilot study.

Of 11 service providers invited to participate from a range of settings, five (3 male, 2 female) expressed an interest in participating and four delivered the intervention: a shared care worker delivering OAT within a GP practice; a nurse delivering healthcare for people experiencing homelessness including through a drugs service setting and outreach in hostels; a community pharmacist providing OAT and NSP within a pharmacy; and a project worker from a women’s hostel. The intervention was delivered within the workplaces of each service provider. Reasons for service providers declining to participate included a lack of capacity, illness, change in role or no response to the invitation.

Each service provider was asked to identify and deliver the intervention, one-to-one to approximately four adults known to be currently injecting drugs or who have injected drugs in the past six months. Service providers were given autonomy to decide who to invite and when to deliver the intervention. The introduction manual stated the toolkit may be useful at various times including:

- During a routine appointment with a client as part of the normal package of support;
- As part of the provision of injecting equipment;
- In response to a client raising an issue or concern about their injecting practice or health (e.g. pain when injecting);
- If a client describes a harmful injecting practice (e.g. inappropriate use of wipes).

Service providers explained the study to potential participants using an information sheet, answered any questions and recorded PWID written informed consent to participate in the intervention and an optional telephone interview after receiving the intervention. PWID received a £20 shopping voucher for participating in the intervention.

Service providers received the intervention manual and were invited to complete online training and to attend an online training session hosted by a harm reduction specialist (DH) and researchers (JK, HF). A recording of the online training was provided to those who could not attend the live session.

Participants receiving the intervention self-completed a socio-demographic questionnaire (e.g. gender, age, previous SSTI, drug use and OAT history) that was provided by the service provider when consent was obtained. Service providers documented the content covered and resources provided. They were also given the option to debrief with JK or DH.

The aim was to deliver the intervention until 20 interviews with PWID had been conducted with JK. Topic guides designed for PWID covered views on the recruitment process, intervention content and purpose, interactions between the service providers and appropriateness of the delivery setting.

Service provider interviews, conducted by JK, explored views on the training and pre-existing knowledge, intervention content and PWID responses to the intervention. All interview participants received a £20 shopping voucher as a ‘thank you’ for their time.

Improvements to the intervention content identified through the interviews were documented in a separate Table of Changes, prioritised according to the MoSCoW criteria and used to further refine the materials.

Thirteen PWID received the intervention and 11 participated in an interview, lasting 38 min on average (range 25–54 mins), between May and November 2021. Most interviews took place on the same day or a couple of days after the intervention, a small number happened later (up to 3 weeks). Most participants were White British (12, 92.3%) and male (8, 61.5%), with a mean age of 43.6 years (SD: 7.0). On average participants had injected for 22.7 years (range 13–44) and most had a history of SSTI (11, 84.6%) (Table 1).

**Table 1:** Participant (intervention recipients) socio-demographic characteristics

Characteristic	n (%)
	N = 13
Ethnicity	
White British	12 (92.3)
Black British	1 (8.7)
Gender	
Male	8 (61.5)
Female	5 (38.5)
Age: years	
Mean (SD)	43.6 (7.0)
Current housing status	
House	7 (53.8)
Hostel	6 (46.2)
Experience of homelessness	
Yes	11 (84.6)
No	2 (15.4)
Length of injecting history	
Years (range)	22.7 (13–44)
Previously attempted to stop injecting	
Yes	13 (100.0)
No	0 (0.0)
Experience of SSTI	
Yes	11 (84.6)
No	2 (15.4)
Currently on opioid agonist treatment	
Yes	12 (92.3)
No	1 (7.7)

All four service providers participated in an interview with a mean length of 51 min (range 42–76 mins).

Interview recordings were transcribed and anonymised. Thematic analysis [29], generating patterns or themes in the data, was undertaken using QSR NVivo. An interpretative approach to qualitative analysis was taken acknowledging the researchers’ active role in the process. Familiarisation with the dataset began by reading the transcripts and coding, line by line inductively and deductively (based on the interview topic guide) by JK. Study researcher HF coded a subset of transcripts to develop and agree the thematic framework which was then used to index and sort the remaining data. As analysis progressed, the coding was refined and restructured to reflect the developing interpretation of the data discussed between JK and HF. Codes and coded data were reviewed to identify patterns of similarity, creating themes and sub-themes related to the research aim. Descriptive and interpretive narrative accounts supported by interview extracts were written up to develop explanations for the patterns within the data.

## Results

### Intervention planning

#### Collating and analysing evidence

##### Review of the relevant literature

The following behaviours were identified in the international literature as related to the risk of developing SSTI among PWID: (i) handwashing/swabbing injection site (place on the body injected into) practice prior to injecting is associated with reduced SSTI [1, 6, 17, 30–32], while an increased risk of SSTI is associated with (ii) vein damage from the use of too much acidifiers for injection preparation [33–35]; (iii) use of non-sterile water [36–38]; (iv) reuse of injecting equipment [11, 31, 32, 38–41]; and; (v) not rotating injection sites [42, 13] (Table 2). In addition, maintaining vein access and minimising pain were identified as important concerns for PWID which can be utilised to enhance engagement with harm reduction interventions [13]. Difficulties accessing veins can be caused by venous sclerosis, hardening and narrowing of veins. Venous sclerosis of peripheral veins can lead to injection into deeper veins which is associated with increased risks of SSTIs such as deep vein thrombosis and ulcers [42].

**Table 2:** Evidence for key behavioural issues and structural barriers the REACT intervention aims to address

Behavioural issue	Key behaviours	Evidence for behaviour	Country	Key finding(s)
Injecting practices contribute to greater risk of developing bacterial skin and soft tissue infections among people who inject drugs	1. Handwashing/swabbing	Larney et al. [1]	International	Four of six studies conducted in England or the USA reported a reduction in skin infections associated with cleaning injection sites; only one of four studies conducted in England or the USA examining handwashing prior to injection found this behaviour to be significantly associated with reduced skin infections
		Vlahov et al. [30]	USA	Of all the persons surveyed, 556/1057 (52.6%) reported cleaning their skin prior to injection at any time and 173/1057 (16.4%) reported cleaning their skin all the time in the 6 months before the interview. The frequency of subcutaneous abscesses was lower among those who reported skin cleaning all the time; a similar trend was noted for frequency of endocarditis
		Murphy et al. [31]	USA	Swabbing the injection site with alcohol before injection was found to have a protective effect against skin and soft tissue abscesses. Significantly fewer people who had developed abscesses, in comparison with controls, had ever used alcohol to clean their skin before drug injection (*p* < 001)
		Dwyer et al. [6]	Australia	Potentially serious or serious injecting-related injuries and disease associated with not always washing hands before injection in the previous 12 months (aOR: 9.3, 2.1–41.8)
		Hope et al. [32]	England	Weak evidence that cleaning injection site every time in the last 4 weeks was associated with a reduced prevalence of injection site infection (OR: 0.6, 0.4–0.8)
		Stein et al. [17]	USA	60% of participants reported ‘rarely or never’ cleaning their skin before injecting during the past 3 months
	2. Use of too much acidifiers	Harris et al. [35]	England	Overuse of acidifiers in injection preparation is common among people who inject drugs in the UK and could play a causative role in venous damage and associated sequelae (skin and soft tissue infection and associated complications). Associations observed between acidifier overuse, femoral injecting and deep vein thrombosis, but not skin and soft tissue infections. Painful injections and damage to peripheral veins were common and often attributed by participants to the use of citric acid
		Ciccarone and Harris [33]	USA and England	Preliminary findings show that different heroin source-forms and preparations have a two-log difference in acidity. Loss of functioning veins (venous sclerosis) is a root cause of suffering for long-term heroin injectors. In addition to perpetual frustration and loss of pleasure/esteem, venous sclerosis leads to a myriad of medical consequences including skin infections, for example, abscesses
		Harris [34]	New Zealand	Opioid injectors in New Zealand using very small amounts of citric acid suffer little vein damage and rarely get skin and soft tissue infections
	3. Use of water	Harris et al. [36]	England	Multiple constraints to sourcing sterile water for injection preparation reported. Participant accounts suggest injection preparation with solvents including puddle water, toilet cistern water, whisky, cola soda and saliva when injecting in public and semi-public spaces. This relates to both behavioural and environmental constraints that increase the risk of infection
		Lloyd-Smith et al. [37]	USA	No strong evidence that using a puddle to inject was a risk factor for developing a cutaneous injection-related infection among people who inject drugs (OR 1.32, 0.83–2.11)
		Hope et al. [38]	England	Higher levels of reported symptoms of injection site infection associated with reusing water to flush syringes (aOR: 1. 28, 1.03–1.59)
	4. Reuse of injecting equipment	Dunleavy et al. [11]	Scotland	Depletion of injecting equipment could lead to reuse of needles, seen as a cause of skin and soft tissue infections by some participants. Needles were reused because of lack of time or inability to replenish supplies due, for example, to weekend closing of convenient needle and syringe programmes or if they woke in the middle of the night. This relates to structural barriers as well as behavioural barriers
		Hope et al. [32]	England	Reporting an injection site infection was associated with cleaning needles/syringes for reuse (aOR:1.5, 1.1–2.1)
		Darke et al. [39]	Australia	Participants who had borrowed used injecting equipment in the preceding month had significantly more current health-related problems at their injecting sites than other participants (3.1 vs. 2.1, t = 3.7, *P* < 0.001)
		Hope et al. [38]	England	Higher levels of reported symptoms of infections were associated with sharing filters in the last four weeks (aOR: 1.31, 0.9–1.59). No strong evidence was found for sharing spoons
		Rance et al. [40]	Australia	75% of participants reported sharing within their partnership. Only one participant reported sharing with someone other than their partner, while eight couples reported never sharing. Of the 26 couples who reported sharing needle–syringes, 20 believed they were hepatitis C virus (HCV) concordant (8 HCV negative and 12 HCV positive) and 14 discordant (8 HCV-positive men and 6 HCV-positive women)
		Murphy et al. [31]	USA	Use of a needle after someone else had used it (*p* = 0.005) and use of a dirty needle (*p* < 0.001) were both significantly more common among cases who reported a skin and soft tissue abscess than among controls
		Wright et al. [41]	England	Participants reported sharing injecting equipment, in particular spoons and filters. Re‐using cleaned needles despite being aware that cleaning may not be effective in reducing the risk of hepatitis C transmission was also identified
	5. Rotating sites	Hope et al. [42]	Bristol	More than half of those surveyed reported having had a ‘missed hit’, and for a quarter this happened at least once a month, with around one in six reporting having a ‘missed hit’ more than four times a month. Those who reported that they had experienced a ‘missed hit’ were twice as likely to also report having had symptoms of injection site infections and injuries
		Harris and Rhodes [13]	England	The facilitation of venous access and care was an initial and enduring rationale for safe injecting practices. Difficult venous access resulted in increased contamination of injecting environments and transitions to femoral injecting. Advice and information on how to avoid venous sclerosis, and how to find and safely access less visible veins, was desired by the majority

Environmental structure	Key structural constraint	Evidence for structural barrier	Country	Key finding(s)
Structural constraints act as barrier to safer injecting practices and contribute to greater risk of developing bacterial skin infections among people who inject drugs	Access to handwashing facilities among homeless people who inject drugs	Harris et al. [36]	England	Funding cuts have impacted not only on housing and welfare provision but access to clean water on the city streets among unstably housed people who inject drugs
		Wright et al. [41]	England	Participants reported injecting in a variety of outdoor public places, while they were homeless, including derelict buildings, back alleys, bushes and underneath bridges
	Citric acid sachet size	Harris et al. [35]	England	Acid sachet size poses a constraint to good practice. The sachet size is a strong signifier of appropriate quantity
	Access to sterile water for injection preparation	Harris et al. [36]	England	Funding cuts have impacted not only on housing and welfare provision but access to clean water on the city streets (e.g. closure of public toilet and increased security in pubs and cafes) among unstably housed people who inject drugs. Drug treatment services, facing sustained budgets cuts of at least 18%, have reduced costs where possible, impacting on the availability of water provision in needle and syringe programme equipment packs
	Access to sterile equipment	McNeil and Small [45]	International	Needle and syringe programmes increase access to material resources and safer injecting education. This is a facilitating factor. Participants expressed understanding that safer environment interventions reduced an array of risks by changing physical and social environments (Kerr et al. 43; Small et al. 44)
	Injecting environment	Dunleavy et al. [11]	Scotland	Participants reported injecting in indoor environments that were unhygienic and higher risk practice when injecting new psychoactive substances. Participants’ experience of skin and soft tissue infections could cause panic and stigma; there was limited knowledge of skin and soft tissue infections prior to first-hand experience
		Wright et al. [41]	England	Participants reported injecting in a variety of outdoor public places, while they were homeless, including derelict buildings, back alleys, bushes and underneath bridges. Participants also reported urgency of injecting outside

There was also evidence that structural constraints act as barriers to safer injecting practices and contribute to greater risk of developing SSTI among PWID. In brief, these included: (i) a lack of access to handwashing facilities when injecting in public spaces [36, 41]; (ii) acidifier sachets containing more than is needed for a single injection [35]; (iii) limited access to sterile water for injection preparation [36]; (iv) lack of access to sterile injecting equipment [45]; and (v) riskier injecting environments including public/semi-public environments [11, 41] (Table 2). These constraints are conceptualised within the ‘risk environment framework’ as the risk of harm from injecting drugs arising from interactions between individuals and their social contexts and environments [46].

##### Co-production with target group representatives and key collaborators

Table 3 demonstrates that target group representatives discussed their injecting practices with key collaborators (DH and CL) openly. They described injecting outdoors or in public spaces as the main barrier to safer injecting practice, with rushed injecting resulting in more opportunities for contamination. An additional structural barrier to safer injecting was unavailability of equipment (e.g. sterile water to prepare injections and post-injection swabs to stem bleeding) from local drug and alcohol services due to cost.

**Table 3:** Key findings from co-production with target group representatives (service providers and people who inject drugs)

Themes	Summary of findings	Action points or intervention development
(i) Service providers		
Acceptability of intervention	All service providers were receptive to the aims of the intervention and expressed willingness to be involved in future research activities to test it with their clients as part of the study	
Professional judgements	Service providers frequently discussed with pride the importance of the relationships they had developed with their clients	Allow service providers autonomy and judgement to decide who and when the intervention is delivered to
	Delivery of the intervention would require a judgement by the service provider as to whether the client would be receptive to the intervention	
	Clients may be aware that their injection practice differs from lower-risk practice. As such, time and appreciation of this, and an understanding that some may not wish to describe their injection practice in detail, is required by service providers	
	Relevance of intervention messages and changes to injecting practices / health seeking behaviour could be increased if delivered to the client at a time of crisis (e.g. presenting with wound site infection)	
Characteristics of target users	Some service providers perceived that an intervention of this type would be most relevant to clients with a shorter injecting history	Allow service providers autonomy and judgement to decide who and when the intervention is delivered to
	Greater barriers to safer injecting practices among clients with less stable lifestyles, long injecting histories and complex social and health needs were noted	
	Openness of clients to discuss injecting practices appeared to differ geographically. Service providers who worked with clients in South Bristol commented that their job role and stigma prevented open discussions and uncertainty as to whether their clients injected or not. This appeared less of a barrier among service providers based in East and Central Bristol. However, it is possible that this is related to the role of the provider as these were not consistent between geographical areas	Develop guidance for service providers to overcome stigma around open discussion about injecting practices
Intervention delivery and training needs	Some service providers had limited time to dedicate to intervention delivery (5–10 min). This could also be influenced by how receptive a client was during an encounter	The intervention should be deliverable in the length of time available to the service provider (5 min upwards)
	All service providers could access a confidential space to deliver the intervention	
	Preferences for intervention training included both face-to-face and online modules	Develop training module that can be delivered face to face or online
(ii) People who inject drugs		
Structural barriers to change	Injecting outdoors presented most barriers to safer injecting practices—rushed, with more opportunities for contamination	Address structural barriers as part of the intervention
	Lack of access to equipment like sterile water to prepare injections and post-injection swabs acted as barriers to safer injecting practices with some people reporting using a range of higher risk water options and either not swabbing or using pre-injection swabs after injecting	
Characteristics of target users	Challenging beliefs of people who have been using drugs long-term with entrenched behaviours is difficult—especially if no history of bacterial infections at wound sites	Encouraging clients to change one key aspect of their injecting practice is most realistic given habits which may have been formed over decades. Training should reinforce to service providers that this may be challenging for their clients
	There was often scope for improving some aspect of the injecting practice. A wide range of different areas for harm reduction strategies were apparent	Given the complexity and range of injecting practices identified as part of the co-production activities, a ‘one size fits all’ approach is not appropriate. Service providers should tailor harm reduction advice specifically to areas identified as more risky following (open) discussion with the client
Delivery of intervention and training needs	Clients may have good knowledge and report ‘best practice’ around injecting behaviours, although this may not always correspond with their history of wound site infections. Images being used as a 'talking point' can help encourage more open discussions, but these may not always be reflective of actual practice.	Service providers should be aware that social desirability bias may impact response from clients. Example questions to probe the client further could be provided as part of the training manual for service providers. Consider involving peers in delivering the intervention, where social desirability is likely to be reduced.
(iii) REACT steering group, academic and clinical experts		
Context to implementation	The main causes of bacterial infections must underpin targets for behaviour change, including: hygiene measures, vein damage, equipment reuse, sharing, not rotating sites, subcutaneous injection, use of water. Stigma and shame are major barriers to overcome in this intervention. Encouraging clients to change one key aspect of injecting practice is most realistic in light of habitual practice which may have developed over decades	Focus on safer injecting practices is required, incorporating reuse of equipment, use of acids, water and rotating sites. Provide resources to promote better hygiene. Service providers should tailor harm reduction advice specifically to areas identified as most risky following open discussion with client. Provide guidance for service providers to overcome stigma around open discussion of injecting practices. Universal messages about preventing infection should be include in the training manual
Scope for supporting change	Attending to the immediate priorities of people who inject drugs, e.g. venous access and care, have the potential to (re)engage clients Small, manageable changes are possible. Structural barriers can be addressed by supporting people to navigate existing structures differently, e.g. provide swabs as part of the intervention. Providers outside of specialist drug treatment services need to be skilled in basic harm reduction practice. Those delivering the intervention need to be knowledgeable and non-judgemental	Frame the intervention around priorities of people who inject drugs. Address some structural barriers, in part, through practical resources to enable safer injecting, e.g. hand sanitiser, swabs with instructions for correct use. Training is required to support service providers to deliver the intervention
Possible targets and strategies for intervention	Delivery to coincide with teachable moments may encourage engagement with the intervention. Structural barriers must be addressed alongside individual-level influences on practice and risk. Focus on one intervention (prevention or treatment) with a narrower focus. Goal setting in this context is problematic as it has the potential for increasing existing stigma associated with drug use, implying blame and judgement on the individual, sense of failure and focuses on individual rather than structural barriers	Address structural barriers (as above.) The focus of the intervention should be on primary prevention of bacterial infections. Do not include goal setting, instead discuss ways to prevent bacterial skin infections, talk about past history and experiences of managing bacterial infections to see if there are any lessons to be learnt and harm reduced in the future.
Presentation, format and framing of intervention	Use images and cards to support engagement, including a range of practices that people who inject drugs relate to and using cards flexibly to open conversations about pain, practices, self-care and seeking treatment. Focus should be on supporting people to care for veins and avoid pain to reduce risk and enable people who inject drugs to prioritise earlier intervention, rather than including a ‘list of things you should do’ which could be stigmatising depending on the mode of delivery. Avoid use of images of infections and focus on primary prevention rather than earlier intervention/ treatment	Develop information to be used alongside the intervention ‘cards’. Frame intervention around vein care to focus on priorities of people who inject drugs

While the target group representatives acknowledged there were areas of injecting practice which could be improved, they felt that challenging the long-held beliefs regarding safe injecting practices among PWID and supporting change of habitual behaviours would be difficult. A knowledge–behaviour gap related to habitual behaviours and structural factors was highlighted with individuals citing good knowledge of ‘best practice’ which they reported to follow although some accounts appeared inconsistent with a history of infections.

Service providers highlighted the importance of existing relationships with PWID and judging whether they would be receptive to the intervention. They suggested that those with a shorter injecting history who were perceived to have less knowledge and behaviours which were less likely to be habitual might be more receptive to the messages in the intervention compared to clients with a longer injecting history. The intervention should also be flexible to meet the limited time available to service providers and how receptive the person is to receiving the intervention. In line with the literature, PWID and key collaborators noted the need to address the structural barriers cited above. PWID also highlighted the need to tailor the intervention to individual risks, and encouragement to change one aspect of injection practice if habits are entrenched. Lastly, key collaborators’ views and perspectives echoed barriers to behaviour change identified in the literature review (Table 2). They highlighted the importance of framing the intervention around the priorities of PWID, particularly minimising pain and supporting vein access to support engagement with the intervention; and the need to avoid stigmatising PWID during the intervention. The latter could be achieved by not using goal setting which our key collaborators highlighted has the potential to increase stigma, a sense of failure and focuses on individual level rather than structural barriers. In support of this, the concept of ‘why try’ in the literature explains that self-stigma—applying and agreeing with stereotypes of oneself—can lead to reduced self-esteem and self-efficacy and discouragement and ability to achieve life goals [47]. In addition, ensuring that the intervention is delivered by trained service providers with sensitivity was viewed as important (Table 3).

### Refinement of the intervention

The prototype intervention focus was realigned and narrowed to primary prevention addressing the following primary causes of SSTI among PWID identified and prioritised in the literature review and consultation activities: (1) poor handwashing and swabbing practice; (2) use of too much acidifiers; (3) use of non-sterile water; (4) reusing injecting equipment; and (5) not rotating sites.

Refining the focus of the intervention also enabled better alignment to the priorities of the target group representatives—‘keeping veins healthier for longer’ and ‘minimising pain’—to maximise engagement with the intervention [13]. Practical resources tailored to participants were added to the intervention materials to support safer injecting practices (e.g. sterile water ampoules, hand sanitisers, wipes with instructions for correct use, Street Injecting Kits (a cardboard box containing injecting equipment and reusable carrier to support separate drug preparation and administration) developed in response to research findings [36]). These materials were supplemented by information resources already developed as required [48].

#### Development of guiding principles

The REACT intervention has three aims: (i) address priorities of PWID by supporting improved vein care and minimisation of pain; (ii) provide appropriate resources to enable less harmful injecting practices and overcome barriers to safer injecting practice at the structural level; and (iii) deliver flexibly to meet the needs of target population (Table 4).

**Table 4:** Guiding principles for REACT intervention

Design objectives that address each key issue	Key intervention features relevant to each design objective
(i) To address priorities of people who inject drugs by making changes to their injecting practices to keep their veins healthier for longer and minimise pain	Provide tailored harm reduction advice to include discussion of the following topics: (i) Environment person injects in (ii) Handwashing/swabbing (iii) Use of acids (iv) Use of water (v) Reuse of equipment (needles, cookers, filters) (vi) Rotating sites Positive, non-judgemental conversation between service provider and client
(ii) To provide appropriate resources to enable less harmful injecting practices and overcome barriers to safer injecting practice at the structural level	Provision of the following resources to help support harm reduction behaviours: Hand sanitisers and wipes (with instructions for use) Injecting tips: #1 bacterial infections Exchange supplies leaflet Injecting tips: #2 prevention and care: abscesses and ulcers Exchange supplies leaflet Injecting tips: #3 staying safe on the street Exchange supplies leaflet Be wise, sterilise poster Exchange Supplies / NIHR ARC West poster Citric acid poster Exchange Supplies: Water ampoules Street injecting kit Citric packets (with instructions for use)
(iii) Flexible approach to delivery of intervention to meet needs of target population	Use of intervention ‘cards’ as appropriate to act as a prompt to discussion on different topics Delivery of shorter version to fit within constraints of appointment time or priorities of client Tailor provision of resources depending on needs of client (e.g. previous experience of bacterial infections; difficulties prioritising safer injecting practice due to dependence; lack of opportunities to follow safer injecting practices; entrenched injecting practices; good knowledge of ‘best practice’; experience of stigma and shame meaning conversations about injecting behaviours are difficult) Intervention delivery should be within the context of a confidential space to facilitate open discussion about stigmatised behaviours Utilise existing relationship between client and service provider to overcome shame in open discussions about behaviours

#### Undertaking a behavioural analysis

The behavioural analysis (Table 5) indicated that the barriers and facilitators to the target behaviours identified and prioritised through the literature review and co-production activities could be addressed through information provision and provision of harm reduction equipment. These REACT intervention strategies relate to three intervention functions from the COM-B model: psychological capability (having the necessary knowledge), reflective motivation (self-conscious planning and evaluation) and physical opportunity (e.g. availability of hand sanitiser). A further five intervention functions (education, training, persuasion, environmental restructuring and enablement) from the Behaviour Change Wheel are used. In turn, these are enacted by six Behaviour Change Techniques: instruction on how to perform a behaviour, information about health consequences, anticipated regret, prompts/cues, pros and cons and restructuring the social environment. For example, a lack of access to handwashing facilities when injecting in public spaces acts as a barrier to handwashing/swabbing target behaviours, which can be addressed through information provision about the importance of handwashing, cleaning surfaces and swabbing the injection site and provision of hand sanitiser and swabs. These strategies relate to psychological capability (COM-B), education (intervention function) and shaping knowledge.

**Table 5:** Behavioural analysis of REACT intervention using the COM-B model of behaviour and Behaviour Change Wheel

Target behaviour	Barrier/facilitator to target behaviour	Intervention strategy	Relevant evidence	Target construct (COM-B)	Intervention functions	Behaviour change techniques^1^
Handwashing/swabbing	Lack of access to handwashing facilities when injecting in public spaces. Lack of swabbing of injection site prior to injecting. Use of swabs to stem bleeding after injecting	Information provision about importance of handwashing, cleaning surfaces and swabbing injection site. Provision of hand sanitiser and swabs	[30] [49]	Psychological capability (knowledge or psychological skills to perform the behaviour) Reflective motivation (self-conscious planning and evaluation) Physical opportunity (environmental support for behaviour)	Education (increasing knowledge or understanding) Training (imparting skills) Persuasion (using communication to induce positive or negative feelings or stimulate action) Environmental restructuring (changing the physical or social contact) Enablement (increasing means/reducing barriers to increase capability (beyond education and training) or opportunity	4. Shaping knowledge 4.1. Instruction on how to perform a behaviour 5. Natural consequences 5.1. Information about health consequences 5.5. Anticipated regret 7. Associations 7.1. Prompts/cues 12. Antecedents 12.5. Adding objects to the environment
Use only necessary amount of acid during injection preparation process	Quantity of acid determined by packet size. Quantity used determined by visual cue of information.	Information provision that excess acid required to dissolve these materials increases injection solution acidity but not psychoactive drug content. Provision of acid sachets with labelling that stresses ‘a whole sachet is far too much for most injections’ during intervention delivery	[35]			
Use of water for injection preparation	Lack of access to sterile water when injecting in public spaces. Measures such as the closure of public toilets and increased security in pubs and cafes have reduced access to clean water for people experiencing homelessness. Sterile water for injection is not included in most injection packs because of local budget constraints.	Information provision about hierarchy of water (intervention delivery and leaflet). Provision of water ampoules/street injecting kits.	[36]			
Minimise reuse of equipment (needles, cookers, filters)	Access to sterile equipment not always available	Information provision about limiting reuse of equipment (needles, filters, spoons) and cleaning of equipment if it is reused. Provision of street injecting kits	[11]			
Rotating sites	Advice and information on how to avoid venous sclerosis, and how to find and safely access less visible veins, was desired by the majority	Information provision about rotating sites. Signposting to specialist healthcare professionals for support to identify veins	[13]			

^1^Behaviour Change Techniques are numbered using the Behaviour Change Technique Taxonomy version 1 [27]

#### Logic model

The logic model details the intervention aim and strategy, alongside the proposed intervention functions and behaviour change techniques and the process and intervention outcomes (Table 6).

**Table 6:** REACT logic model

Intervention aim	Intervention strategy	Intervention functions and behaviour change techniques	Process outcomes	Intervention outcomes
To reduce bacterial infections among people who inject drugs by: (i) Making changes to injecting practices to keep veins healthier for longer and minimise pain (ii) Providing appropriate resources to overcome structural barriers to safer injecting practice (iii) Being flexible in approach to delivery of intervention to meet needs of target population	Training of service providers Staff time and expertise Private/confidential space for brief motivational interview during appointment Intervention materials: Intervention ‘cards’ and resources to facilitate conversation about safer injecting practices	Education: Instruction on how to perform a behaviour Training: Instruction on how to perform a behaviour Persuasion: Information about health consequences Anticipated regret	Number of organisations in which the intervention is delivered. Number of organisations and individuals who received training to deliver the intervention. Number and length of appointments delivered by each service provider. Number of people who inject drugs who did not attend appointment (reach) or refused offer of taking part and reasons (e.g. competing priorities, illness). Content covered in each appointment (e.g. handwashing/swabbing, use of acids) Intervention resources provided during appointment (e.g. hand sanitiser, sterile water, information leaflets)	Primary outcome: Reduction in development of bacterial infections (people who inject drugs) Mechanisms of change: Acceptability of intervention delivery and materials (service providers and people who inject drugs) Increase in knowledge/understanding of safer injecting practices to keep veins healthier for longer and reduce pain (service providers and people who inject drugs) Increase in confidence to support people who inject drugs to use drugs more safely (service providers) Increase in safer injecting practices to keep veins healthier for longer and reduce pain (people who inject drugs)
	Environment restructuring: Resources to support behaviour change (e.g. hand sanitiser, sterile water, information leaflets)	Environmental restructuring: Prompts / cues Adding objects to the physical environment		

Context				
National policies, initiatives and campaigns; local policies, initiatives and campaigns; impact of COVID-19 pandemic; social norms and values; professional norms and values; and organisational policies and procedures, structural barriers to safer injecting				
People who inject drugs who access a range of services may have: previous experience of bacterial infections; difficulties prioritising safer injecting practice due to dependence; lack of opportunities to follow safer injecting practices (e.g. injecting outdoors); entrenched injecting practices; good knowledge of ‘best practice’; and experience of stigma and shame meaning conversations about injecting behaviours are difficult. Attending to immediate priorities of people who inject drugs has potential				

### Intervention optimisation

#### Design and refinement of intervention materials

The amended intervention was created as a set of themed cards addressing risk factors for SSTI using a design brief, written content and suggested accompanying images.

The cards included a title page with the aim of the intervention, suggested ways of using the cards to facilitate a positive, non-judgemental conversation and an overview of the themes. The intervention cards and introduction manual (available for free download here: https://express-licences.bristol.ac.uk/product/react-reducing-bacterial-infections-materials) and training course are available on the Exchange Supplies website [50]. The intervention training was supplemented with pre-existing Exchange Supplies e-learning for NSP practitioners [51].

Overall, PWID and key collaborators provided positive feedback on the optimised designs. Suggested alterations centred around using appropriate, clear language (e.g. ‘part used amp’ (ampoule) was changed to ‘part used sterile water’) and clear imagery (e.g. ensuring an image of an acidifier could not be confused with a transparent plastic bag used for drugs and using colour coding to indicate the gradient of risk with water options). The depiction of masculine hands was perceived to be potentially alienating for women who inject drugs. This was addressed in the next iteration of the designs by including female hands alongside male.

#### Intervention pilot

##### Process outcomes

The mean intervention delivery time was 24 min (range 15–40 min) (Table 7). All intervention themes were covered in every session. Leaflets related to prevention of SSTI were the most frequently provided resource (n = 11), followed by antimicrobial handwipes (n = 10) and sterile water ampoules (n = 10) (Table 7).

**Table 7:** Process outcomes for delivery of the REACT intervention

Delivery time (mins)	24 (15–40)
Content covered in each session	n (%)
Handwashing/hygiene: Yes	13 (100.0)
Use of acids: Yes	13 (100.0)
Use of water: Yes	13 (100.0)
Reusing equipment: Yes	13 (100.0)
Rotating sites: Yes	13 (100.0)
Resources provided*	N
Leaflet: Injecting tips: #1 bacterial infections	11
Leaflet: Injecting tips: #2 prevention and care	11
Leaflet: Injecting tips: #3 staying safe on the street	11
Poster: Be wise, sterilise	1
Leaflet: Citric acid	6
Clinell antimicrobial handwipes	10
2 ml Water for injections	10
Street injecting kit	9
Sterile citric acid sachets	5

* Missing data assumed as not provided

Three themes, ‘intervention acceptability’, ‘intervention delivery’ and ‘intervention outcomes’, are reported below with illustrative quotations for participants (PWID receiving the intervention) and service providers delivering it.

##### Intervention acceptability

Service providers’ and PWID accounts suggested that PWID responded well to the intervention and viewed it positively. There were no dissatisfactory or negative aspects of participating in the intervention disclosed to the researcher during the interviews, though some intervention topics were more relevant than others. For example, rotating sites was less relevant to those who had been injecting for longer and had lost vein access to some sites. One service provider also suggested there was too much talking and information accompanying the toolkit which could affect how much is absorbed.

PWID viewed the purpose of the intervention as encouraging safer injecting and supporting behaviour change. The focus on vein care and reducing pain was viewed as important, relevant issues for PWID who had current or previous experience of injecting-related health problems and were motivated to avoid future infections.

The cards that [service provider, organisation 3] was reading out to me and explaining to me, some of the things I was like, ‘Oh, I didn't know that’. Yeah, it’s useful information, and it’s good to know different things, because it's about trying to be safe, and for me as well trying to reduce getting abscesses and stuff. (…) I thought I had my way of doing it and I felt safe, when really it’s not. Participant 13, Organisation 3

A gap in information provision from credible sources and opportunities to have these conversations was highlighted, especially for people not accessing specialist drug services. Participants talked about learning their injecting practice from peers rather than professionals and viewed this intervention as a good opportunity to learn new information:

All they knows is what they seen – they don’t really know it from the professionals, and what could happen if you didn’t do that – how it burns you, like acid, and dirty water is infected and stuff (…) Loads of things I’ve got from it [REACT intervention]. Participant 1, Organisation 1

The intervention was seen as trying to help people through positive messaging to improve the experience of using drugs, rather than punitive measures:

The resource works really well in terms of language because the toolkit and the language of the person that’s delivering it is about helping with pain, so reducing pain, improving the life of them, that kind of stuff so it’s really positive messages rather than negative. Don’t do that because if you do that this will happen, it’s more positive than that. Service provider, Organisation 1

The content and design of the toolkit were generally viewed positively, with agreement that the topics were appropriate and the short messages and imagery were easy to understand:

Because it’s got pictures on it as well as information so you can see things, what you’ve done wrong, like the water thing. Like it says not to use hot water, use coldish water. If you can, use one of them snap top bottles. If you can’t the next one is water out of the kettle. (…) So I think the cards are quite a good idea as well. Participant 4, Organisation 2

The resources provided as part of the intervention were also viewed positively and were considered to support safer injecting practices.

Suggestions for improvements to the content included providing more detailed harm reduction information on how to inject safely and adding bullet points to the back of each card with key points to support delivery. Small changes to the design of the materials were suggested (e.g. highlighting key words, using more contrasting colours, re-ordering information for ease of understanding, simplifying the designs so they are less busy and key information stands out, checking the language used is plain English). Providing PWID with the intervention cards to take away was proposed to reinforce the messages. Additional information explaining how to use the resources (e.g. the water ampoules) would also be helpful.

##### Intervention delivery

Service providers described a range of approaches, typically opportunistic, to inviting PWID to receive the intervention. Considerations were made around their relationship with the individual, the likelihood they would attend an appointment and a judgement about whether it was appropriate to invite them at that time.

Interviewer: Could you tell me a bit more about how you approach people? And how you invited them?

I judged it by how they approached the counter (...), just from body language to see if they were in a rush. If they didn’t seem too rushed, then I asked, ‘Do you have some time to speak to me? Service provider, Organisation 4

PWID expressed a range of reactions to the invitation. Two PWID were surprised to be approached due to the person delivering the intervention (i.e. not expecting that individual to be delivering the intervention), the number of people with whom the service provider worked and because they were trying to stop using drugs:

I know [name]’s got probably about 120 people on his list so, to be honest, I was quite shocked that he’d rung me, but me and my friend have actually been trying to sort ourselves out, do you know what I mean, to come off all this. Participant 5, Organisation 2

Reasons given by PWID for participation included: not being in a rush; no objections to participating; wanting to contribute to research or to do something positive; interest in helping to prevent others from having injecting-related problems; invitation style/service provider demeanour; interest in learning; relevance of the intervention to the individual and viewing the intervention as potentially helpful.

I’ve not long got out of hospital myself with an abscess. (…) If you’re doing something wrong in the process or just like catch up every couple of year or something. (…) Yeah, just try and find out how I got it and how not to get it again. Participant 4, Organisation 2

PWID also viewed incentives to encourage participation positively.

Service providers suggested reasons for PWID non-participation including work commitments, time constraints and a perception that people who refused lacked interest in learning about harm reduction. Arranging a time to do the intervention could also be challenging and time-consuming, due to competing priorities related to sourcing and buying drugs.

PWID felt the service providers had sufficient knowledge to deliver the intervention and their existing relationship meant they felt comfortable with them. To some extent, the latter reflects the service providers choice of who they delivered the intervention to. The service providers’ confidence to discuss the topics within the toolkit varied, which appeared to relate to perceptions of their existing knowledge, previous experience in delivering harm reduction advice and communication style used within their professional role. The training provided was viewed positively by service providers and as important in supporting successful delivery. There was some preference among the PWID interviewed for service providers with experiential knowledge of drug use to deliver the intervention:

I know he’s [service provider, organisation 2] been there himself and done it. So it’s not like someone who’s read it out of a book or read it off a card. He’s actually been there and done it, like, knows the symptoms and he’s had things wrong with him like I’ve had things wrong with me and stuff like that even. Off someone like him, it’s believable. Participant 4, Organisation 2

However, participants appreciated that the service providers without lived experience were open to learn from the participants and were honest about their knowledge limitations.

Generally, service providers tailored the intervention to topics of interest/relevance to the individual, identified through conversation about the individual’s injecting practices and knowledge of the intervention content, to judge which topics to discuss in the most detail. Two participants did not talk through all the cards in order, instead card selections were made according to their interest and what was most relevant. Indeed, one provider gave the participants the cards to decide which to talk about. One provider decided to go through all the cards because the content overlapped and they were all viewed as important topics; however, they would go into less depth depending on the topic’s relevance.

Some people who, for example, wash their hands well, I would just briefly show them the picture of ‘This is where you miss washing your hands.’ So, I didn’t have to talk about it too much. Things, like for example, the different types of water to be used, if they showed that they didn’t have much knowledge in that area, then I would go into a bit more depth. Yes. I mentioned everything, but I suppose didn’t go into detail into every single thing. Service provider, Organisation 4

I would say, “Talk me through your injecting practices, where are you? What are you using, where are you getting your water from?” I didn’t want to prompt them to say cold water is better, I didn’t want them to think there was a right and wrong answer so I kind of got them to give me a general overview step by step of what they would be doing wherever they were when they were injecting and I’d start off like that. So to pinpoint then which areas we needed to focus on. Service provider, Organisation 3

Both service providers and PWID described the style of delivery as conducive to discussing injecting practices openly (e.g. showing compassion, being non-judgemental, and phrasing questions sensitively). This contrasted with previous negative experiences of interacting with health professionals. Participants appreciated service providers being open and interested in understanding their experiences and recognising them as experts. Additionally, using themed cards to facilitate conversation was felt to facilitate non-judgemental, two-way conversations, rather than a didactic delivery of information, which allowed participants to share their experiences of injecting in a non-threatening/confrontational way:

Using tools and resources that are visual because it really helps in the engagement process. It’s less threatening, gives you something to focus on. So, you know, it’s a good way of learning. It’s a good way of having conversation. It’s less conflict. It’s kind of more collaborative. (…) Where you’re sitting can make a big difference, so if you’re working with somebody with a resource you’re usually sitting alongside them. I mean, just the word sitting alongside, that’s wonderful, (…), it just creates a better environment, a less threatening environment and it creates an opportunity for the person to talk rather than to be talked at or given information. It allows them to lead rather than be told where to go. They can take the cards themselves and just go through themselves. Service provider, Organisation 1

The intervention setting—confidential, private spaces—was generally viewed positively by PWID. The intervention length was felt to be appropriate and acceptable to PWID. However, two service providers indicated their capacity to deliver the intervention at a greater scale was limited:

When I came out there were a lot of tasks and patients waiting for me to do things. A lot of our consultations with people, they don’t last 20, 30 minutes for someone looking at a rash, or something like that. So, this was quite time consuming. Service provider, Organisation 4

Participants suggested that individuals who had recently had an infection or people who were newer to injecting would benefit most from the toolkit:

A lot of the younger ones don’t know too much about it. That’s one of the reasons why all that would be fantastic, for them to know straightway, because it would make it a lot more easier and safer. Participant 6, Organisation 2

There were mixed views across interviewees regarding future group delivery raised as a suggestion during the interviews. While some viewed this as beneficial in creating an environment to learn from others, others felt one-to-one delivery allowed people to discuss their personal experiences more easily.

#### Participant responses to the intervention

Most PWID reflected that they had learnt at least some new information through the intervention:

I learned a lot of things yesterday that I didn’t know. It was good that I got involved, I’m glad that I got involved now because I know how to bang up properly now. Participant 14, Organisation 3

Some PWID accounts suggested that the intervention is a useful reminder of safer injecting practices.

Participation led to reflections on injecting history and practice and health-related issues. The intervention appeared to help PWID make sense of injecting-related problems such as finding vein access difficult and being ‘prone to’ abscesses:

I only just found out today from [service provider] that I was putting too much citric in for instance and I destroyed my veins I think after that way cos I was using a whole sachet per £10 worth of heroin and I’ve just been told now that’s probably the worst part of my injection was the overuse of citric and I didn’t know what I was doing and I didn’t have this REACT training do you know what I mean? If I’d had it before I probably would have veins in my arms still. Participant 10, Organisation 4

Most PWID accounts indicated intentions to make small changes or refinements towards safer injecting practice as outlined within the intervention content such as rotating injection sites more, using sterile water, using less citric acid, using swabs before injecting and not using swabs afterwards to stem bleeding. One PWID felt confident that the intervention would help reduce their risk of abscesses.

I’m going to not use the whole pack of citric, I’m just going to use a tiny little bit (…) because I’ve always seen people put the whole pack of citric in. Participant 14, Organisation 3

I’m not using the sterets [wipes] anymore to put on my injection site afterwards because it bleeds out and that’s what I was doing, if I didn’t have any tissue, I’d use one of the sterets like to put on the injection site. Participant 12, Organisation 3

Other participants did not anticipate making changes to their injecting practices as they felt that they already followed best practice. For instance, adding a small amount of citric acid at a time.

Despite some positive intentions, the interviews revealed difficulties prioritising harm reduction practices. Withdrawal symptoms also made it difficult to follow harm reduction practices especially if these practices meant risking losing drugs (e.g. if too much citric acid has been used), making it more difficult to find a vein or taking longer to successfully inject (e.g. trying a new injecting site). Ease, speed and success were prioritised:

Rotating the sites, she did talk to me about that, and I do rotate, I do go in different places in my legs, but like I said to you just now, I said to [service provider, organisation 3] that to be honest, if I’m withdrawing or whatever, or even if I'm not, I just want to try and get my hit the quickest and the easiest way. Participant 13, Organisation 3

She said you know they say you know a whole sachet is probably too much but it’s always good to add more if you need it but you can’t take away if you put too much in. Especially if you’re ill and you’re withdrawing and (…) you’ve now got 20 quid in the syringe or in the dish but you put too much citric in you’re either going to have to use it or you’re going to have to try and raise up 20 quid when you’re ill and that’s hard to do when you’re ill you know and the last thing you want to do is ruin the drugs you’ve got. Participant 10, Organisation 4

It was therefore important to be sensitive when delivering the intervention to the habitual nature of injecting and sense of safety in injecting in the same way:

It’s kind of working really collaboratively with that person and curious (…). They’ve spent their whole life feeling ashamed and embarrassed and humiliated and you’ve got to be really careful with how you play things out and so getting as much information from them can be really helpful because then you can give lots of positive reinforcement and positive encouragement alongside some harm reduction. (…) So it’s about (…) the way you communicate things and the way you challenge behaviours because a lot of these behaviours are… they’ve been habitual behaviours for years. (…) They feel really safe in the ritual of it and dangerous or risky behaviours can be really, really hard to challenge. Service provider, Organisation 1

Continued structural barriers to safer practices include availability of injecting equipment resulting in reuse or sharing and unhygienic injecting environments.

## Discussion

We report the development, acceptability and feasibility of a novel behavioural intervention. Intervention development adopted an evidence-based and iterative approach, incorporating co-production and engagement with PWID, service providers and key collaborators.

The initial prototype intervention focused on targeting individual-level behaviour change around prevention and treatment of SSTI. Using the Person-Based Approach to engage with target group representatives, we identified that factors within the socio-physical environment increase the risk of injecting-related harm and need to be addressed. This broadened the focus beyond an individualised approach.

The REACT intervention seeks to go some way to addressing calls for interventions focusing on the priorities of PWID such as stigma (i.e. delivery of the intervention in a non-judgemental, stigmatising manner), pain [11], venous access and care [9, 13]. The intervention appeared to be acceptable to the PWID and service providers who participated in the pilot and was mostly feasible to deliver in the settings where it was trialled in Bristol. The intervention content and design and the materials given out to support harm reduction were viewed positively. These findings may be partly explained by who participated in the pilot and the relationship between service providers and participants. In line with the REACT intervention logic model, there were some indications that PWID had increased knowledge and intended to make changes to their injecting behaviours, though barriers to safer injecting practice remained prominent. PWID described increased awareness of the risks of using swabs post-injection, and although rotation of sites was not always viewed as possible, others considered trying other injection sites following the intervention. Importantly, despite its sensitive nature, discussing site rotation was acceptable.

The intervention delivery length (mean 24 min) and content delivered (all themes were discussed) suggest that the intervention was not delivered within brief interactions as outlined within the guiding principles. This did not appear to affect perspectives on the value of the intervention. However, delivery within brief interactions could address the issue of service provider capacity and support wider-scale implementation in relation to the time and costs of delivery, particularly within busy community pharmacy settings. Service providers may have wanted to test the intervention fully for the purpose of the research, viewed all aspects of the intervention as important and relevant, or this could have been unclear in the training. Key changes proposed to the intervention included small changes to the design which have been implemented using the MoSCoW prioritisation process, additional information for PWID on safer injecting and for service providers delivering the intervention and additional information about the resources provided to support their use. In relation to the finding that some topics were perceived as less personally relevant to more experienced PWID, in these circumstances, the intervention could be framed around dissemination of the intervention to peers in the participant’s network.

An ongoing challenge for public health researchers is determining when scientific evidence, such as the findings of the current study, is sufficient for further research to establish effectiveness. The academic team considered potential study designs to establish the effectiveness of the REACT intervention. This was weighed against the potential benefits of enabling the intervention considered at low risk of causing harm (as supported by the small-scale pilot) to be made available and used in practice more rapidly. While randomised controlled trials (RCTs) are widely accepted as the strongest form of evidence (after systematic reviews), trials can be scientifically challenging to undertake within the field of public health due to their inherent complexity. It was therefore decided by the project team that wider implementation of the REACT intervention to other settings outside Bristol could support conversations around safer injecting practices with the potential to enhance knowledge about prolonging vein access, reducing pain and preventing SSTI [13]. To achieve a larger-scale rollout and to enable appropriate capacity to be available for delivery, organisational-level adoption of the intervention may be needed. This intervention will also be added to existing guidance for commissioners and providers of drug services in England to support services to be ‘wound aware’ [52]. Following a news story and targeted dissemination to relevant key stakeholders, organisations, and networks in March 2023, there were  ~170 downloads in  August 2023 of the final version of the intervention materials from the University of Bristol online shop (https://express-licences.bristol.ac.uk/product/react-reducing-bacterial-infections-materials). Downloads have been made by individuals across a range of organisations including local authorities, drug and alcohol services and charities, public health agencies, national health and mental health trusts, housing support organisations and charities and academics. Future follow-up of those downloading the materials could help understand wider implementation experiences. By taking a more pragmatic approach to wider implementation, we were able to make the REACT intervention resource available more rapidly and without the inherent costs and length of time required to undertake an RCT.

### Comparison to previous literature

Previous research and consultation with stakeholders informing this intervention confirms the importance of including themes beyond hygiene (e.g. reducing use of acid) [9]. Others have also found that swabs are not always used as intended (e.g. post-injection) [11]. Our findings agree with qualitative work in Scotland which also found that participants reported infrequent injection site rotation pre- or post-SSTI to ensure successful injection [11]. In contrast, others have shown that a number of PWID do rotate their sites [13]. In our study, loss of vein access was one explanation for not rotating sites.

In line with previous evidence [35], the REACT intervention aimed to support individual-level behaviour change and address structural/environmental influences. For instance, the intervention included practical resources tailored to participants to support safer injecting practices (e.g. sterile water ampoules, hand sanitisers, wipes with instructions for correct use, Street Injecting Kits [36]) and information resources [48]. This approach was intended to avoid further stigmatisation of PWID and is in line with the ‘risk environment framework’ [46]. Given the continued constraints on safer injecting highlighted in this study, such as difficulties prioritising safer injecting and environmental constraints, one approach to enhancing REACT’s effects could be to combine it with interventions more directly addressing these issues such as interventions that improve housing and living standards [53], OAT provision (to reduce the effects of withdrawal on safer injecting) and community-based overdose prevention centres (OPC). Over 130 OPCs (including an unsanctioned centre in Scotland running between 2020 and 2021) are available in approximately 14 countries and provide safe environments for people to use drugs with support from trained professionals who can offer evidence based interventions including naloxone and psychosocial support [54]. Observational studies have shown that OPCs can reduce self-reported high-risk injecting practices [55], and therefore, interventions like REACT could help further support this outcome.

There are limited interventions, especially in the UK, to prevent SSTI among PWID, and therefore, this study adds to the evidence base. An intervention in France delivered by trained harm reduction programme staff comprising direct observation of injecting practice, identification of harmful injecting practice and discussion of safer injecting practices reduced the likelihood of unsafe injection practices (e.g. sharing injecting equipment) and injection site complications (e.g. abscesses) and increased the likelihood of safer injection practices (e.g. handwashing) at 12 months [14, 15]. In contrast, REACT was designed to be accessible to staff outside specialist harm reduction settings supporting staff to have brief conversations with PWID. Direct observation of injecting practice was therefore not viewed as feasible or appropriate within such diverse and time limited settings.

Another intervention in France [16] co-designed with stakeholders and PWID and delivered within harm reduction programmes in one or more sessions using enablement (provision of alcohol hand sanitiser), education to increase knowledge and understanding of hand hygiene and training (information on appropriate hand sanitising technique) demonstrated acceptability of education and hand sanitiser provision, good adherence to the steps of hand sanitiser use and low rates of adverse reactions. This study found evidence for increased self-reported hygiene and a reduction in injection-related complications at six-week follow-up [16]. Findings from focus groups also indicated that those living in unstable housing and young PWID compared to older PWID (who were expected to find changing their injecting practice more difficult) were perceived to benefit most from the intervention. The latter is in line with the current study’s findings that less experienced PWID were expected to benefit most from the intervention. In contrast to this study’s focus on hand hygiene, REACT targeted multiple behaviours associated with SSTI which potentially supports greater harm reduction and scope to tailor to individual needs.

The SKIN intervention in the USA [17, 18, 56] delivered in inpatient hospital units increased the likelihood of skin cleaning prior to injection, but did not reduce SSTIs significantly. Reduced injection-related hospital visits compared to the control group were found but this did not lead to a significant reduction in total hospital visits or hospitalisations [17, 18]. The SKIN intervention consisted of a baseline assessment of handwashing, injection site cleaning and injecting equipment cleaning. Participants were asked to demonstrate best practice for these behaviours. The observed practices were then scored on a scale developed for the study. Intervention participants received education on injecting-related infections and information and demonstrations of effective handwashing, injection site cleaning and cleaning needles. Participants were asked to practice these behaviours. The risk assessment completed at baseline was then discussed and used to develop an individualised ‘risk reduction change plan’. Participants received a workbook containing goals and information and clean injecting equipment. A follow-up session involved reviewing the risk reduction change plan, discussion of participants progress towards meeting their goals and barriers experienced. The plan was then updated with new goals as needed. Similar to REACT, this intervention was tailored to the individual and provided participants with resources to help support safer injecting practices. A strength of the SKIN intervention is the demonstration and practicing of hand, injection site and injecting equipment cleaning. The follow-up session also allows an opportunity to discuss behaviour change and support further changes. REACT could also be delivered over multiple sessions where practical and within hospital settings during admission for SSTI. Indeed recent hospitalisation for SSTI and interest in learning how to prevent future infection were given as reasons for participation in the current study, indicating that this may be an opportune time for delivery. To support this, barriers to accessing hospital health care need to be addressed including judgement and stigma, delayed provision of OAT to manage withdrawal symptoms and fear of punitive and life changing treatment (e.g. amputation) [10]. The iHOST study, co-produced by people who use opioids, has developed a multicomponent intervention including an e-training module to help reduce stigma against people who use opioids, and to provide hospital staff with communication approaches [57].

### Strengths and limitations

Strengths of our approach to intervention development include the use of an established research methodology, the Person-Based Approach [24], and the co-production activities with PWID, service providers and key collaborators using an iterative approach throughout to determine the design of the intervention. To ensure we developed an intervention that was underpinned by theory, we used constructs from the COM-B model and Behaviour Change Wheel [26] to define the intervention processes and components and behaviour change techniques [27] to be targeted. However, the review of the evidence base is not fully comprehensive of the literature.

Although behavioural interventions like REACT are inexpensive to deliver, recruiting service providers was challenging which may reflect recognised pressures across the drug and alcohol services sector. Disinvestment in drug and alcohol treatment and recovery services have led to a reduction in workforce capacity [58]. A further limitation is that the online training for service providers was not mandated and completion was not formally assessed, though interviews indicated it was completed.

Recruitment of PWID was lower than anticipated, in part, due to service providers’ capacity. The views expressed by contributors may not be generalisable to PWID in different geographical locations, and there may be different views among PWID at different stages of their injecting history. Those who participated in the intervention may also differ from the broader population of PWID in terms of their existing positive relationships with service providers, age, experience of SSTI and length of time injecting.

The sample reflects the ageing population of PWID in the UK. PWID were selected by service providers who felt they could deliver the intervention to them in part due to their existing relationship, which was highlighted as important in our intervention development work. However, this means we do not know how a universal offer of the intervention would be received. It is also possible that participants’ positive responses to the intervention and minimal suggested improvements reflect social desirability bias because of their relationship with the service providers. The researcher conducting the interviews tried to mitigate this by emphasising the importance of providing honest feedback to improve the intervention and probing for negative views.

Service providers who were willing to take part may differ in attitudes towards PWID and receptiveness to harm reduction interventions than those who did not volunteer. Successful scale up of the intervention may depend on identifying others with similar positive attitudes or require additional training and support to encourage delivery. Structural barriers to lower-risk injecting practice remain, and we acknowledge that although addressed as far as possible by this intervention, there are a range of additional issues that this intervention cannot address (e.g. lack of safe spaces to inject in).

Development of the intervention was time intensive, involving multiple collaborators, and we cannot comment on the resulting acceptability or feasibility of the wider implementation. Findings from this small-scale intervention pilot, delivered to 13 PWID by four service providers, within one UK city should therefore be interpreted with caution. Adjustments may be required to deliver the intervention in other geographical locations accounting for different contexts.

## Conclusions

Using the Person-Based Approach, we have gained insight into the psychosocial context of the target population and optimal design features by using an iterative approach to intervention development and integrating feedback at each stage of intervention development. This allowed us to adapt features of the intervention in anticipation of likely intervention usage to increase persuasiveness and feasibility to deliver the intervention in practice. The intervention was acceptable and motivated positive intentions for some PWID around safer injecting practice. This intervention could be implemented and evaluated further as part of a wider package of interventions.

## Data Availability

Due to the sensitivity of the data involved, these data are published as a controlled dataset at the University of Bristol Research Data Repository data.bris, at https://doi.org/10.5523/bris.y6pp0w6oacmx2s3mkda3qqstc. The metadata record published openly by the repository at this location clearly states how data can be accessed by bona fide researchers. Requests for access will be considered by the University of Bristol Data Access Committee, who will assess the motives of potential data reusers before deciding to grant access to the data. No authentic request for access will be refused, and reusers will not be charged for any part of this process.

## Abbreviations

PWID: People who inject drugs
REACT: REducing bActerial infecTions
